# Circulating Plasma miRNA and Clinical/Hemodynamic Characteristics Provide Additional Predictive Information About Acute Pulmonary Thromboembolism, Chronic Thromboembolic Pulmonary Hypertension and Idiopathic Pulmonary Hypertension

**DOI:** 10.3389/fphar.2021.648769

**Published:** 2021-05-28

**Authors:** Alexandre Todorovic Fabro, Juliana Machado-Rugolo, Camila Machado Baldavira, Tabatha Gutierrez Prieto, Cecília Farhat, Flavia Regina Rotea ManGone, Sabrina Setembre Batah, Heloísa Resende Cruvinel, Maiara Almeida Aldá, Jhonatas Sirino Monteiro, Adriana Inacio Pádua, Sirlei Siani Morais, Rogério Antônio de Oliveira, Marcel Koenigkam Santos, José Antônio Baddini-Martinez, João Carlos Setubal, Claudia Aparecida Rainho, Hugo Hyung Bok Yoo, Pedro Leme Silva, Maria Aparecida Nagai, Vera Luiza Capelozzi

**Affiliations:** ^1^ Department of Pathology, Laboratory of Histomorphometry and Lung Genomics, Faculty of Medicine, University of São Paulo, São Paulo, Brazil; ^2^ Department of Pathology and Legal Medicine, Respiratory Medicine Laboratory, Ribeirão Preto Medical School, University of São Paulo (USP), São Paulo, Brazil; ^3^ Health Technology Assessment Center (NATS), Clinical Hospital (HCFMB), Medical School of São Paulo State University (UNESP), Botucatu, Brazil; ^4^ Laboratory of Molecular Genetics, Center for Translational Research in Oncology, Cancer Institute of São Paulo (ICESP), São Paulo, Brazil; ^5^ Bioinformatic Laboratory, Institute of Chemistry, University of São Paulo (USP), São Paulo, Brazil; ^6^ Pulmonary Hypertension Care Center, Department of Internal Medicine, Ribeirão Preto Medical School, University of São Paulo (USP), São Paulo, Brazil; ^7^ Department of Biostatistics, Plant Biology, Parasitology and Zoology, Institute of Biosciences, São Paulo State University (UNESP), Botucatu, Brazil; ^8^ Department of Chemical and Biological Sciences, Institute of Biosciences, São Paulo State University (UNESP), Botucatu, Brazil; ^9^ Pulmonary Hypertension Care Center, Department of Internal Medicine, Botucatu Medical School, São Paulo State University (UNESP), São Paulo, Brazil; ^10^ Laboratory of Pulmonary Investigation, Carlos Chagas Filho Biophysics Institute, Federal University of Rio de Janeiro, Centro de Ciências da Saúde, Rio de Janeiro, Brazil; ^11^ National Institute of Science and Technology for Regenerative Medicine, Rio de Janeiro, Brazil; ^12^ Department of Radiology and Oncology, Medical School of São Paulo State University (UNESP), São Paulo, Brazil

**Keywords:** microRNA, RNA-sequencing, blood plasma, pulmonary hypertension, thromboemboilc disease

## Abstract

Idiopathic pulmonary artery hypertension (IPAH), chronic thromboembolic pulmonary hypertension (CTEPH), and acute pulmonary embolism (APTE) are life-threatening cardiopulmonary diseases without specific surgical or medical treatment. Although APTE, CTEPH and IPAH are different pulmonary vascular diseases in terms of clinical presentation, prevalence, pathophysiology and prognosis, the identification of their circulating microRNA (miRNAs) might help in recognizing differences in their outcome evolution and clinical forms. The aim of this study was to describe the APTE, CTEPH, and IPAH-associated miRNAs and to predict their target genes. The target genes of the key differentially expressed miRNAs were analyzed, and functional enrichment analyses were carried out. The miRNAs were detected using RT-PCR. Finally, we incorporated plasma circulating miRNAs in baseline and clinical characteristics of the patients to detect differences between APTE and CTEPH in time of evolution, and differences between CTEPH and IPAH in diseases form. We found five top circulating plasma miRNAs in common with APTE, CTEPH and IPAH assembled in one conglomerate. Among them, miR-let-7i-5p expression was upregulated in APTE and IPAH, while miRNA-320a was upregulated in CTEP and IPAH. The network construction for target genes showed 11 genes regulated by let-7i-5p and 20 genes regulated by miR-320a, all of them regulators of pulmonary arterial adventitial fibroblasts, pulmonary artery endothelial cell, and pulmonary artery smooth muscle cells. AR (androgen receptor), a target gene of hsa-let-7i-5p and has-miR-320a, was enriched in pathways in cancer, whereas PRKCA (Protein Kinase C Alpha), also a target gene of hsa-let-7i-5p and has-miR-320a, was enriched in KEGG pathways, such as pathways in cancer, glioma, and PI3K-Akt signaling pathway. We inferred that CTEPH might be the consequence of abnormal remodeling in APTE, while unbalance between the hyperproliferative and apoptosis-resistant phenotype of pulmonary arterial adventitial fibroblasts, pulmonary artery endothelial cell and pulmonary artery smooth muscle cells in pulmonary artery confer differences in IPAH and CTEPH diseases form. We concluded that the incorporation of plasma circulating let-7i-5p and miRNA-320a in baseline and clinical characteristics of the patients reinforces differences between APTE and CTEPH in outcome evolution, as well as differences between CTEPH and IPAH in diseases form.

## Introduction

Acute pulmonary thromboembolism (APTE), chronic thromboembolic pulmonary hypertension (CTEPH) and idiopathic pulmonary artery hypertension (IPAH) are pulmonary vascular diseases, that can reveal a pathophysiological continuum. On the other hand, they are rather different pulmonary vascular diseases in terms of clinical presentation, prevalence, pathophysiology, and prognosis. Acute pulmonary thromboembolism is characterized by acute obstruction of the pulmonary artery usually leading to pulmonary artery hypertension (PH), being the third most common cause of acute cardiovascular death (Douma et al., 2010; Goldhaber et al., 2012; Konstantinides et al., 2014). However, due to the non-specific clinical symptoms and signs, the diagnosis of APTE is often missed or delayed (Kostadima and Zakynthinos, 2007). CTEPH, a complication of APTE and the fourth types of PH, is characterized by the remodeling of the pulmonary arteries by fibro-organized tissue Connell et al., 2015 (Simonneau et al., 2018). The incidence of CTEPH in APTE survivors is about 3–9%, and 2 years survival in untreated patients with a mean pulmonary artery pressure greater than 50 mmHg was as low as 10% (Simonneau et al., 2018). However, the early recognition of CTEPH progression is difficult due to the gradual beginning and absence of effective biomarker (Guo et al., 2014; Miao et al., 2017). IPAH is a rare but life-threatening cardiopulmonary disease of unknown etiology, with progressive loss of quality of life, showing a 5 years overall survival rate of 50% (Berger et al., 2011; Simonneau et al., 2018; Koudstaal et al., 2020). IPAH characterizes by progressively increased pulmonary artery pressure and pulmonary vascular resistance, and up to now, there is no specific treatment (Santos-Ferreira et al., 2020; Zhang et al., 2020). Thus, comprehensive analyses of potential mechanisms with possible implications in time of evolution and disease form in APTE, CTEPH and IPAH are urgently needed. Interestingly, they share common pathophysiology changes, including fibrinolysis, pulmonary artery endothelial cell (PAEC) proliferation; pulmonary artery smooth muscle cell (PASMC) proliferation, migration, and contraction; inflammation; and pulmonary arterial adventitial fibroblasts (PAAF) proliferation, activation, and migration. Therefore, there should be some differences between these three pulmonary vascular diseases at molecular level, especially microRNAs (miRNAs).

MicroRNAs (miRNAs) are small, non-coding endogenous RNA molecules consisting of approximately 20 nucleotides that suppress gene expression post-transcriptionally by binding to the ‘‘seed sequences’’ in 39 untranslated regions (UTRs) mRNAs (Chen et al., 2008; Kosaka et al., 2010). Identified as stable cell-free miRNAs in serum or plasma, circulating miRNAs are discharged passively and selectively to blood by various cells, and may play as receiver or messenger in cell communication (Kosaka et al., 2010). Through illness, miRNAs in the sick cells are released into the circulation, and the circulating miRNA profile is adopted with the disease characteristics (Chen et al., 2008). Therefore, circulating miRNAs have been widely explored as potential blood-based biomarkers for individual cardiovascular diseases diagnosis. Furthermore, it is also reasonable to assume that identification of molecular pathways and axon guidance enriched by differentially expressed miRNAs target genes and interactions between them provide a molecular basis for the unbalance between hyperproliferative and apoptosis-resistant phenotype particularly in the three principal pulmonary artery cell involved including PAEC, PASMC, and PAAF (Graham et al., 2010; Chen and Jiang, 2018; Yuan, 2019; Santos-Ferreira et al., 2020). Thus, we hypothesize that analyzing different circulating plasma miRNAs in APTE, CTEPH and IPAH, we can describe the early APTE evolution to CTEPH, as well as irreversible fibro-cellular narrowing of pulmonary arteries in diseases form, including CTEPH and IPAH.

This study aimed to describe the APTE, CTEPH, and IPAH-associated miRNAs. miRNAs differentially expressed in APTE, CTEPH, and IPAH samples, compared with healthy samples were identified, and the target genes were predicted. The target genes of the key differentially expressed miRNAs were analyzed, and functional enrichment analyses were carried out. The miRNAs were detected using RT-PCR. Finally, we incorporated plasma circulating miRNAs in baseline and clinical characteristics of the patients to detect differences between APTE and CTEPH in time of evolution, as well as differences between CTEPH and IPAH in diseases form.

## Methods

### Diagnosis and Clinical Characterization of Patients and Healthy Subjects

The Research Ethics Committee of Botucatu Medical School, Botucatu, Brazil (CAAE: 27704714.4.0000.5411) approved this study, and all participants provided their written informed consent for study participation under the Declaration of Helsinki. This study is reported in accordance with the Strengthening the Reporting of Observational Studies in Epidemiology (STROBE) reporting guideline.

The New Generation Sequencing (NGS) cohort was composed of 19 APTE, 14 CTEPH, 14 IPAH patients and 13 healthy subjects (CTR). For RT-PCR confirmation, 5 APTE, 6 CTEPH, 6 IPAH patients and 4 healthy subjects were included. The patients were recruited over approximately 3 years (from 2015 to 2018) from the Pulmonary Hypertension Center and the Emergency Room of University Hospital of Botucatu Medical School (FMB-UNESP, Botucatu, Brazil). To participate of the study, healthy subjects should present normal tricuspid annular plane systolic pressure (TAPSE) by echocardiogram. In patients with APTE, the study was performed with less than 48 h in relation to the acute episode. Only CTEPH patients with distal thrombotic lesions participated in the study. Reported characteristics of patients were assessed at diagnosis moment. Hemodynamic and clinical classification of idiopathic pulmonary hypertension was done according to the 2018 World Symposium on Pulmonary Hypertension (Simonneau et al., 2019). Patients with CTEPH were inoperable and managed with targeted IPAH therapy according to the 2018 guideline (Simonneau et al., 2019).

Table 1 summarizes the baseline and clinical characteristics of our NGS cohort and healthy subjects.

**TABLE1:** Baseline and clinical characteristics of the NGS cohort and healthy subjects.

Characteristic	CTR *N* = 13	APTE *N* = 19	CTEPH *N* = 14	IPAH *N* = 14	*P-*value^a^
Age (yr. Median and interval quartile)	42 (32–52)	55 (39–71)	53 (42–68)	45 (25–65)	0.85
Gender (female/%)	11/84%	17/89%	10/71%	11/78%	0.07
Race category					0.90
*White*	11/84%	13/68%	7/50%	10/71%	
*Black*	2/16%	6/32^&^	7/50%	4/29%	
Smoking	0	3/16%	4/28%	1/7%	0.20
DVT (positive/total)	0	1/5%	3/21%	2/14%	0.13
mPAP (mmHg), mean ± SD	–	–	25.2 ± 13.4	43.3 ± 12.3^&^	**0.02**
sPAP (mmHg), mean ± SD	–	–	75.4 ± 9.4	89.2 ± 8.6^#^	**0.01**
PVR (dyn s cm^−5^)	–	–	336 ± 2.48	586 ± 5.38^&&^	**0.009**
Oxygen saturation, mean ± SD	–	95.42 ± 2.4	90.92 ± 8.07	89.42 ± 6.22^##^	**0.001**
Respiratory rate, mean ± SD	–	20.89 ± 5.34	18.64 ± 3.02	19.14 ± 3.48	0.05
Heart rate, mean ± SD	–	92.05 ± 18.38^†^	81.71 ± 15.19	78.78 ± 11.53	**0.001**
CI (L min^−1^ m^−2^)					0.05
*>4*	–	–	12/85%	5/36%	
WHO functional class					**0.002**
*>2*	–	–	9/14^†^	5/14	
TAPSE (mm)	16.9 ± 0.4	17.6 ± 0.6	15.1 ± 1.3	14.2 ± 1.5^###^	**0.001**
NTproBNP (pg/ml)*1	–	74.6 ± 4.2	182 ± 7.7^&&&^	47 ± 5.3^####^	**0.001**
CRP (mg/L)*I	–	17.33 ± 2.35^††^	6.14 ± 1.34	4.2 ± 1.2	**0.009**
D-dimer (µg/L)	–	8,161.86 ± 1729.89^†††^	217.78 ± 92.12	–	**0.001**

Abbreviations: CTR, controls; APTE, acute pulmonary thromboembolism; CTEPH, chronic pulmonary thromboembolism with pulmonary hypertension; PAH, idiopathic pulmonary arterial hypertension; DVT, deep venous thrombosis; mPAP, mean pulmonary arterial pression; sPAP, systolic pulmonary arterial pressure; PVR, pulmonary vascular resistance; CI, cardiac index; TAPSE, tricuspid annular plane systolic excursion; NTproBNP, N-terminal pro-brain natriuretic peptide; CRP, C-reactive protein.

^a^The Mann–Whitney U test, two-sample Kolmogorov–Smirnov test were used to examine differences between two groups.

Chi-square test were used to examine differences between categorical variables. Multiple comparison was done by non-parametric Kruskall–Wallis test. & = mPAP different between CPTEH and PAH; # = sPAP different between PAH and CPTEH; && = PRV different between CPTEH and PAH; ## = oxygen saturation different between CTR and PAH;† = Heart rate different from CTR; † = WHO Functional Class different between CPTEH and PAH; ### TAPSE different from PAH to CPTEH and APTE; &&& = NTproBNP different from CTR, CPTEH and PAH; †† = CRP different from CTR, CPTEH and PAH; ††† = D-dimer different between APTE and CPTEH.

Bolded values are statically significant.

### Blood Plasma Collection and RNA Isolation

Blood samples (6 ml each) were collected into EDTA anticoagulant tubes and mixed by upside-down rotation ten times. After a two-step centrifugation process (1,100 g at 4°C for 10 min, followed by 1,300 g at 4°C for 20 min), the plasma was transferred into rnase/DNase-free tubes and stored at −80°C until further analysis. We obtained RNA plasma using the miRNeasy Serum/Plasma kit (QIAGEN, Hilden, Germany) according to the manufacturer’s instructions. The RNA quantity and integrity were determined by Qubit^®^ 3.0 Fluorometer (Invitrogen, Life Technologies, CA, United States) and the Agilent 2100 Bioanalyzer (Agilent Technologies, Santa Clara, CA, United States), respectively.

### Library Preparation and High-Throughput Sequencing

Small RNA library was built with TruSeq Small RNA Library Prep kit (Illumina Inc, San Diego, CA, United States) following the manufacturer's recommendations. We transcribed the RNA to cDNA and amplified it using a common primer and a primer containing the barcode index sequences. This cDNA was separated by PAGE 6%. Libraries was recovered via purification and quality checked on Bioanalyzer 2100 (Agilent Technologies). Library concentration was confirmed (>2 nmol/L) by qPCR using the KAPA Library Quantification kit (KAPA Biosystems). Indexed small RNA libraries were multiplexed in equimolar amounts, denatured, and loaded on flow-cell lanes for sequencing. All samples were sequenced at the Institute of Biotechnology (IBTEC-UNESP, Botucatu, Brazil) using the Illumina NextSeq 500 according to the manufacturer’s instructions consisting of 36bp single-end reads in 50 cycles.

### Bioinformatic Analysis

#### Screening of Differentially Expressed miRNAs

Small RNA sequencing raw data was translated into a raw FASTQ sequence one. Then, we filtered the FASTQ format reads to remove rows with low quality via adaptor sequences and sequences inclusion with a quality score <20, using Fastx-Toolkit (http://hannonlab.cshl.edu/fastxtoolkit/). We aligned the filtered reads against the reference human genome, version hg19, using Bowtie v2.0.6. Then, the reads from each individual were merged to identify mature miRNAs using miRDeep2.0.0.8. In detail, the reads were mapped and quantified using the miRDeep2 mapper. pl module and quantifier. pl module, respectively, with standard parameters. Mature miRNAs were downloaded from mirbase v21 (http://www.mirbase.org). The differentially expressed miRNAs (DEmiRNAs) between the samples were normalized and identified by the Bioconductor/R DESeq2 package using ten counts per miRNA as a detection threshold. The criteria for microRNA expression level measuring was CPM (counts per million) value, and we adjusted *p* values to control the false discovery rate (FDR). We identified significant DEmiRNAs using log2 (fold change) >1 and adjusted FDR.

#### Functional Enrichment Analysis for Differentially Expressed miRNAs

Gene Ontology (GO) and Kyoto Encyclopedia of Genes and Genomes (KEGG) were used to assess the functional annotation of the DEmiRNAs and their enrichment in different biological pathways, respectively. Functional annotation and miRNAs pathways were performed using the DIANA-miRPath v3.0 database according to the published instructions (Vlachos et al., 2015). The Gene Ontology (GO) and Kyoto Encyclopedia of Genes and Genomes (KEGG) were used to assess the functional annotation of the DEmiRNAs and their enrichment in different biological pathways, respectively. The functional annotation and miRNAs pathways were performed using the DIANA-miRPath v3.0 database according to the published instructions (Vlachos et al., 2015).

#### Construction of the Differentially Expressed miRNAs-Gene Regulatory Network

In order to predict the DEmiRNA target genes (Hamberg et al., 2016), we used the miRTargetLink (www.ccb.uni-saarland.de/mirtargetlink) to predict the DEmiRNA target genes (Hamberg et al., 2016). Then, we calculated and visualized the mirtarbase and miRanda interaction network information (Enright et al., 2003; Chou et al., 2016). Thus, we only considered the validated targets with strong evidence. The Venn diagram indicating the intersected targets genes was generated by a Draw Venn Diagram online tool (http://bioinformatics.psb.ugent.be/webtools/Venn/).

### Quantitative Reverse-Transcription PCR Confirmation

For quantitative real-time reverse transcription PCR (qRT-PCR) confirmation, we selected the miRNAs with the highest significant differences in expression revealed by next-generation sequencing experiment. Then, we performed the quantification of miRNA expression using miScript miRNA PCR primer assays and miScript SdYBRGreen PCR kit (Qiagen, Valencia, CA, United States) on a StepOnePlus™ Real-Time PCR System (Applied Biosystems, Foster, United States) following the manufacturer’s instructions. Finally, we calculated each miRNA levels using the comparative cycle threshold method (ddCT method) relative to the mean of *miR-16* and *miR-483*. All samples were performed in triplicate.

### Statistical Analysis

The chi-square or Fisher’s exact test were performed to assess differences in clinical variables. In addition, General Linear Model was applied to evaluate the associations between miRNAs and clinically related parameters. The statistical software program IBM SPSS (version 22; Armonk, NY, United States of America) performed all analyses. *p* value < 0.05 was considered as statistically significant.

## Results

### Screening of Differentially Expressed miRNA

**FIGURE 1:**
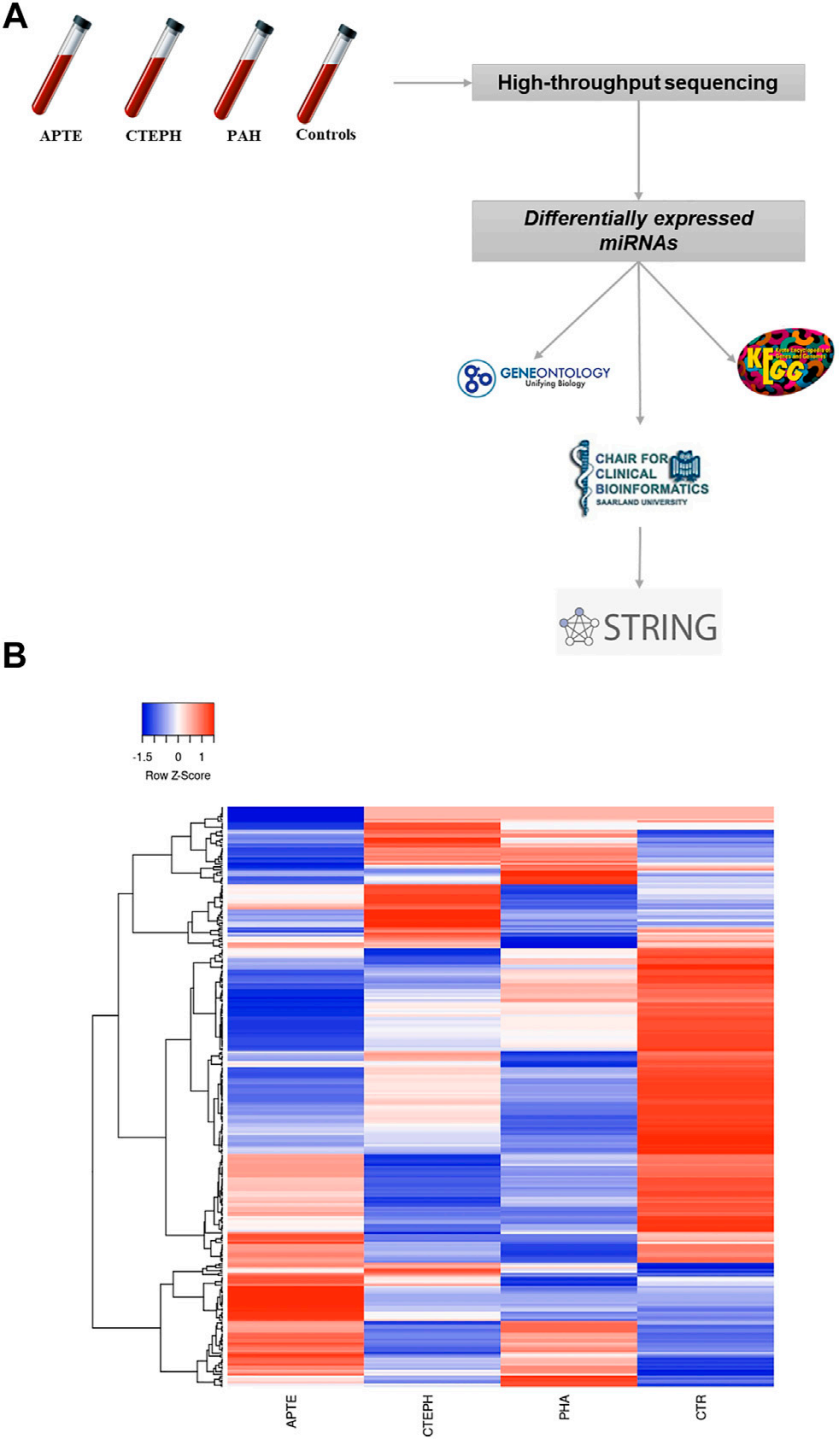
**(A)** Flowchart showing the strategy for detection of differentially expressed miRNAs through next-generation technology. **(B)** Hierarchical clustering of the four experimental groups based on their miRNA expression patterns (*p* adj <0.05, |log 2 (foldchange)| > 1) filtering of next-generation sequencing results. 299 miRNAs were subjected to hierarchical clustering by average linkage method calculating with Pearson distance. Green and red colors represent increased and decreased expression levels (transformed into *z*-scores), respectively, when compared with the average level of each miRNA. Columns and lines represent samples and miRNA, respectively. APTE, acute pulmonary thromboembolism; CTEPH, thromboembolic pulmonary hypertension; PAH, idiopathic pulmonary hypertension; and CTR, Controls.

**FIGURE 2:**
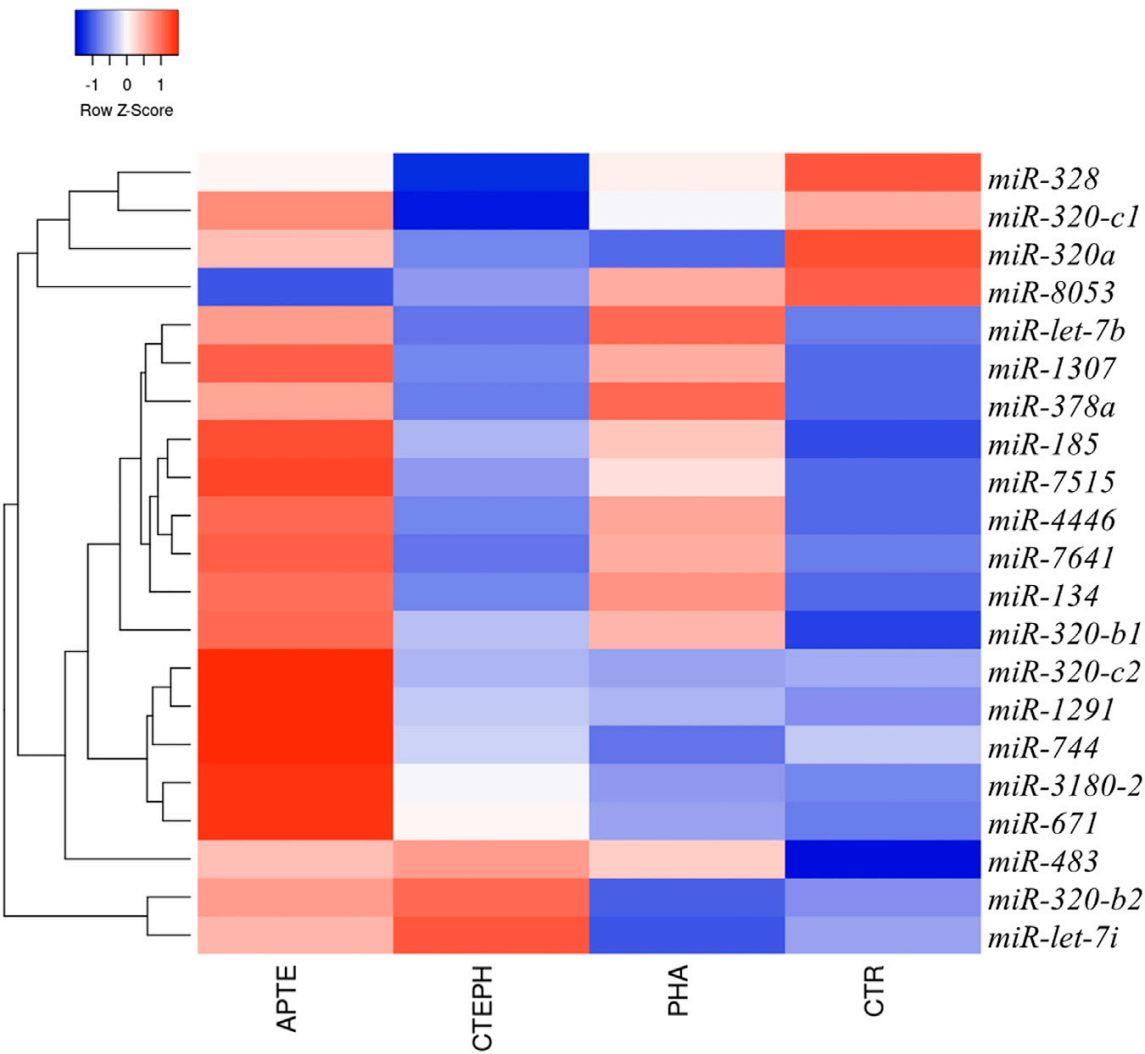
Circulating miRNAs signature in APTE, CTEPH, PAH patients, and healthy controls. Hierarchical clustering and heatmap diagram of the optimum circulating miRNAs from APTE (*n* = 7), CTEPH (*n* = 8), PAH (*n* = 5), and healthy controls (*n* = 2). Green and red colors represent increased and decreased expression levels (transformed into *z*-scores), respectively, when compared with the average level of each miRNA. Columns and lines represent samples and miRNA, respectively. APTE, Acute Pulmonary Thromboembolism; CTEPH, Chronic Thromboembolic Pulmonary Hypertension; PAH, Pulmonary artery Hypertension; and CTR, Controls.

Overall, 299 known mature miRNAs were identified in our NGS cohort according to the QC inclusion criteria (Supplementary Table S2; Figures 1A,B). Among these, a total of 21 differentially expressed miRNAs were obtained according to the cut-off criteria (*p* adj <0.05, |log 2 (foldchange)| >1) (Supplementary Table S2). The heat map of these differentially expressed miRNAs is shown in Figure 2.

### Target Gene of Differentially Expressed miRNA Prediction Analysis

**FIGURE 3:**
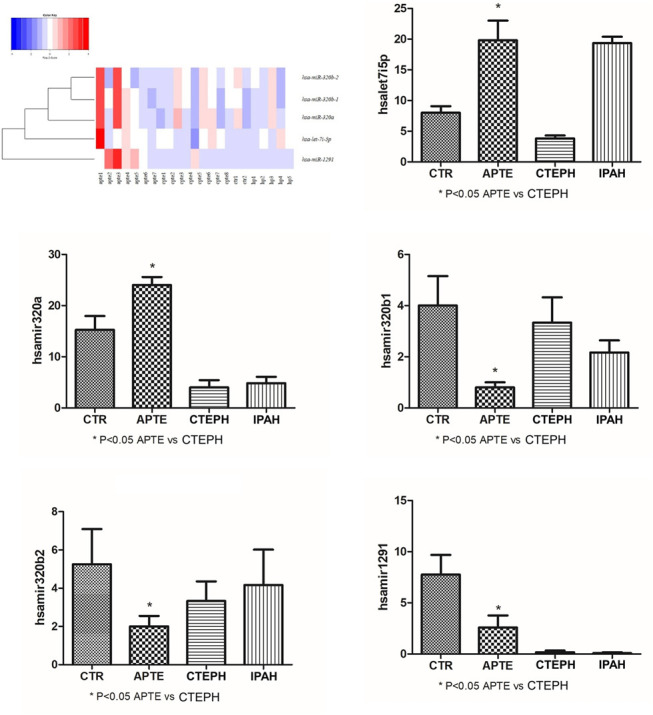
Heatmap and box plots graphs representing the five miRNAs—*let-7i-5p*, *miR-320a*, *miR-320b-1*, *miR-320b-2*, and *miR-1291* significantly associated with the APTE group compared to the CTEPH group after testing adjustment by FDR (Benjamini–Hochberg). Red and blue colors represent increased and decreased expression levels (transformed into *z*-scores), respectively, when compared with the average level of each miRNA. APTE, acute pulmonary thromboembolism; CTEPH, thromboembolic pulmonary hypertension; PAH, idiopathic pulmonary hypertension; and CTR, Controls.

The top five results for the number of target genes regulated by differentially expressed miRNAs are shown in Figure 3. Of the 21 miRNAs, let-7i-5p, miR-320a miR-320b-1, miR-320b-2, and miR-1291 regulated the most target genes. The heatmap represents all of them (Figure 3). The five miRNAs-let-7i-5p, miR-320a, miR-320b-1, miR-320b-2, and miR-1291 were up-regulated in APTE and, with exception of miR-320b-1, down-regulated in CTEPH (Table 2 and Figure 3). In IPAH, we found miRNA-320a and miR-1291 down-regulated and up-regulation of let-7i-5p and miR-320b-1 (Table 2 and Figure 3).

**TABLE 2:** miRNAs with ID starting with “ENSG” are known miRNAs.

Id	logFC	PValue	FDR
ENSG00000199179	3.16	0.00023	0.02525
2_dna:REF_16,302	4.53	0.00024	0.2525
ENSG00000211543	4.26	0.00025	0.2525
ENSG00000208037	4.50	0.00028	0.2525
ENSG00000281842	5.21	0.00071	0.04868
ENSG00000221406	4.29	0.00082	0.04868

The only one with a different ID “2_dna: REF_16,302” is a likely new miRNA. LogFC represents how many times the miRNA is expressed in the APTE group in relation to CTEPH. FDR is a statistical method for correcting the *p*-value for multiple tests.

### Functional Enrichment Analysis for Differentially Expressed miRNAs

**FIGURE 4:**
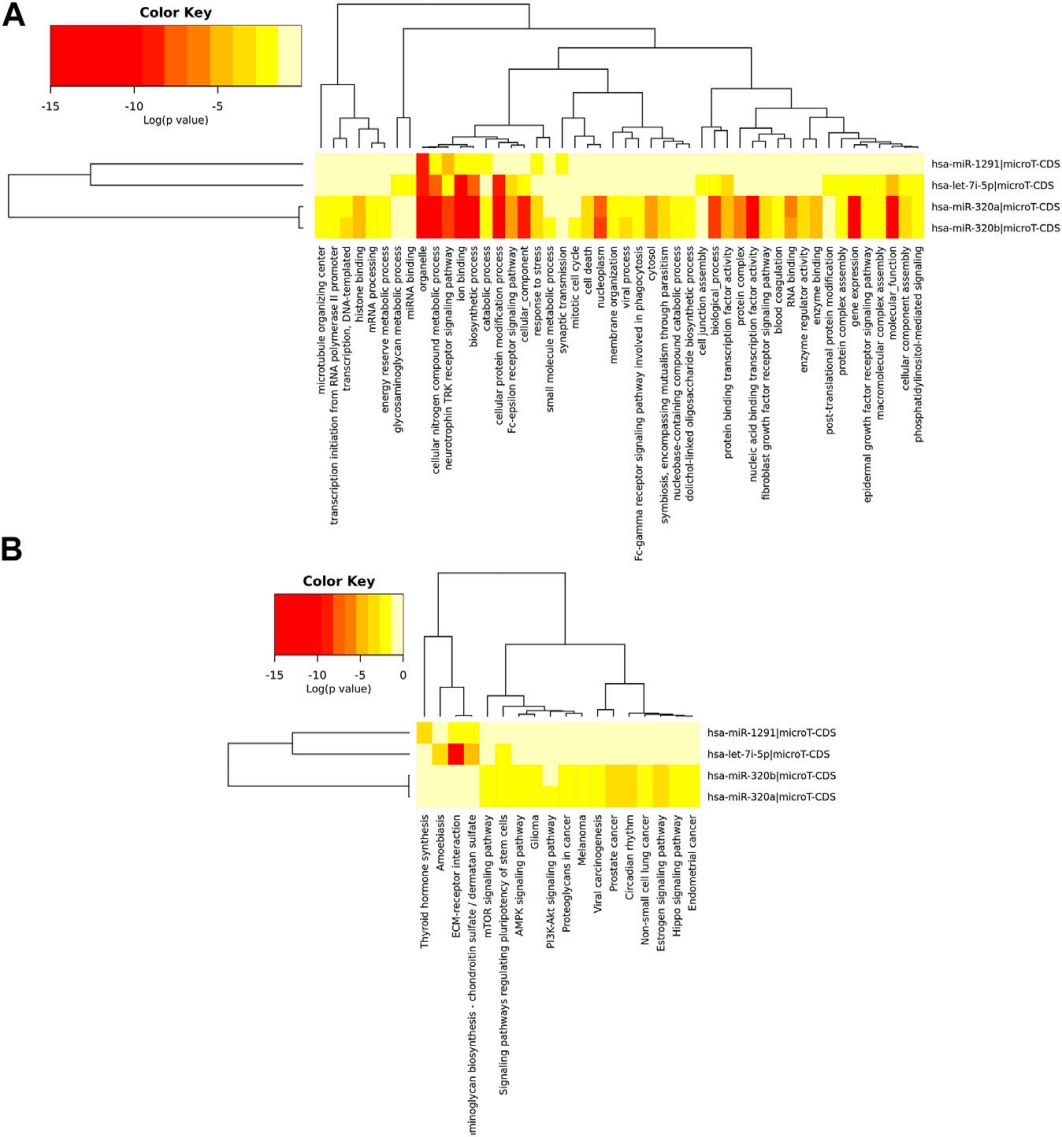
Hierarchical clustering based on predicted target genes of upregulated miRNAs in APTE samples. **(A)** Ontology-based hierarchical clustering of genes identified as predicted targets of upregulated miRNAs in APTE. Using mirPath from DIANA tools, the upregulated miRNAs APTE samples were used to prepare a heatmap based on the gene ontology of predicted target genes. **(B)** Pathways based hierarchical clustering of predicted target genes as part of upregulated miRNAs in APTE samples. Kyoto Encyclopedia of Genes and Genomes (KEGG) based mapping of putative target genes in various pathways as part of the upregulated miRNAs and their canonical target genes.

As shown in Supplementary Tables S1–S3 and Figure 4, the upregulated miRNAs in APTE (let-7i-5p, miR-320a, miR-320b-1, miR-320b-2, and miR-1291) and CTEPH (miR-320b-1) were significantly enriched in pathways of cancer, glioma, and PI3K-Akt signaling pathway, whereas the downregulated miRNAs in CTEPH (let-7i-5p, miR-320a, miR-320b-2, and miR-1291) and IPAH (miR-320a and miR-1291) were mainly enriched in pathways of cancer and apelin signaling pathway. Among them, let-7i-5p and miR-320a were significantly enriched in pathways of cancer and axon guidance.

### The Network Construction for Target Genes Regulated by Differentially Expressed miRNAs

**FIGURE 5:**
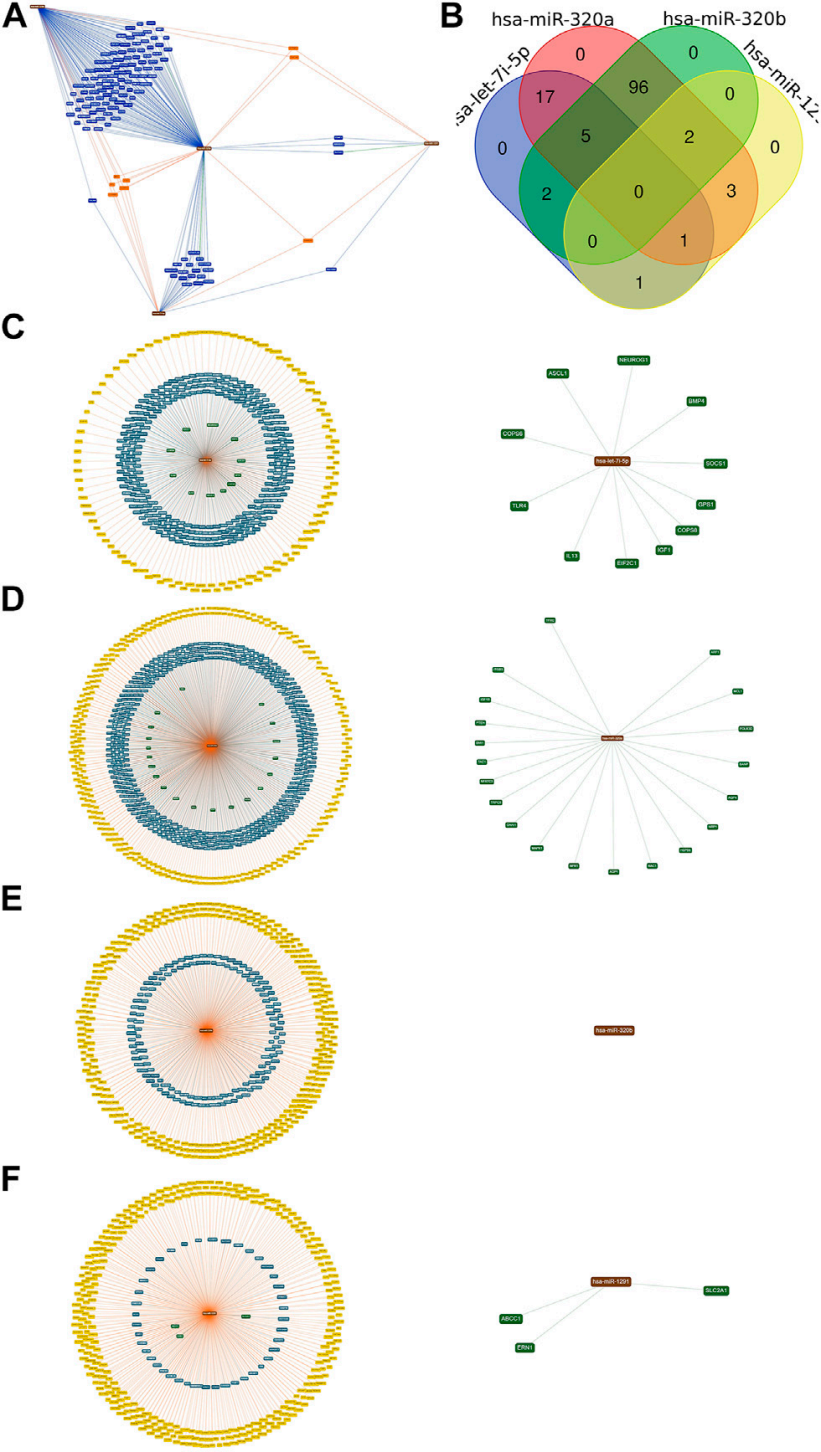
The target and predicted genes of the upregulated miRNAs in APTE samples **(A)** The bioinformatics tool miRTargetLink Human executed the network. **(B)** The Draw Venn Diagram online tool generated the Venn diagram. **(C)** The central node represents the *hsa-let-7i-5p*. **(D)** the *hsa-mir-320a*. **(E)** the *hsa-mir-320b*; and **(F)** the *hsa-mir-1291* surrounded by the validated target with predicted targets in yellow, weak (blue), and strong (green) evidence, which was highlighted in circles.

We constructed the miRNA-Target network for the differentially expressed miRNAs. MiRTargetLink found 126 genes targeted by two or more of the selected microRNAs (Figure 5A). We performed the Venn diagram to indicate the possibly targeted intersected genes upregulated in APTE, down-regulated in CTEPH and IPAH (Figure 5B). Finally, we evaluated the potential targets for each miRNA. The *let-7i-5p* presented 11 possible targets: *IL13, EIF2C1*, *TLR4*, *COPS8*, *NEUROG1*, *BMP4*, *ASCL1*, *COPS6*, *IGF1*, *GPS1*, and *SOCS1* (Figure 5C). The *miR-320a* showed 20 targets: *MAPK1*, *TAC1*, *RAC1*, *GNAI1*, *HSPB6*, *BMI1, AQP4*, *NPR1*, *ITGB3*, *ARF1*, *AQP1*, *NRP1*, *MCL1*, *TRPC5*, *IGF1R*, *POLR3D*, *NFATC3*, *TFRC, PTEN*, and *BANP* (Figure 5D). There were no strongly evident targets for *miR-320b-1/b-2* (Figure 5E). Lastly, *miR-1291* possibly targeted the *SLC2A1*, *ERN1*, and *ABCC1* genes (Figure 5F).

### Functional Enrichment Analysis of the Target Genes of the Key miRNAs

The target genes regulated by upregulated differentially expressed miRNAs in APTE (let-7i-5p, miR-320a, miR-320b-1, miR-320b-2, and miR-1291) and CTEPH (miR-320b-1) were mainly enriched in 11 GO terms (Supplementary Table 1S) and 5 KEGG pathways (Supplementary Table 3S), and the target genes regulated by downregulated differentially expressed miRNAs in CTEPH (let-7i-5p, miR-320a, miR-320b-2, and miR-1291) and IPAH (miR-320a and miR-1291) were mainly enriched in 40 GO terms and calcium signaling pathway. Androgen receptor (AR), a target gene of hsa-let-7i-5p and has-miR-320a, was enriched in pathways in cancer. Protein kinase C Alpha (PRKCA), also a target gene of hsa-let-7i-5p and has-miR-320a, was enriched in 14 of 18 KEGG pathways, such as pathways in cancer, glioma, and PI3K-Akt signaling pathway.

### Detection of miRNAs Using RT-PCR

**FIGURE 6:**
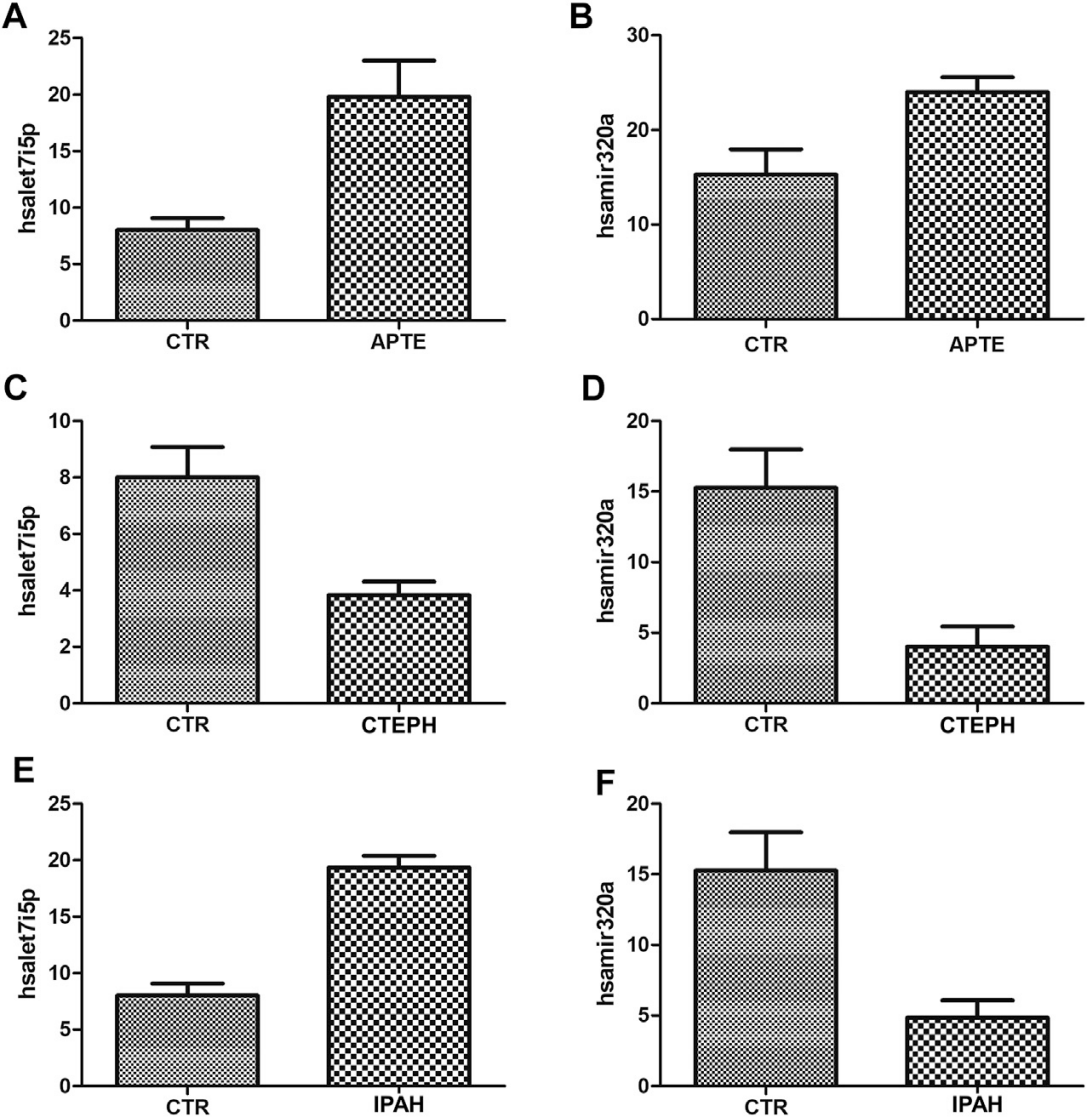
**(A)–(F)** Plot graphs of the expression of miRNAs in APTE, CTEPH and IPAH samples compared with that in control samples by qRT-PCR. *p < 0.05 (Independent-Samples Mann–Whitney test). APTE group *n* = 5; CTEPH (*N* = 6), IPAH (*N* = 6) and CTR (*N* = 4). APTE, acute pulmonary thromboembolism; CTEPH, chronic thromboembolism pulmonary hypertension; iPAH, idiopathic pulmonary hypertension; CTR, healthy subjects; qRT-PCR, Quantitative Reverse-Transcription.

The expression of let-7i-5p and miRNA-320a in APTE samples was significantly higher than that of the control samples (*p* = 0.01 and *p* = 0.02, respectively; Figures 6A,B). In contrast, the expression of let-7i-5p and miRNA-320a was significantly lower in CTEPH compared to the control samples (*p* = 0.02 and *p* = 0.01, respectively; Figures 6C,D). IPAH samples presented significant higher expression of let-7i-5p than control samples (*p* < 0.01; Figure 6E) and significant lower expression of miRNA-320a compared to the control samples (*p* = 0.02; Figure 6F).

### Baseline and Clinical Characteristics of Patients and Healthy Subjects

Table 1 summarizes the baseline and clinical characteristics of patients and healthy subjects. Patients and healthy subjects presented a similar distribution of age, gender, race, and tobacco history. Deep venous thrombosis was detected in six out of 47 (13%) patients. In patients who underwent right cardiac catheterization, the level of mean pulmonary artery pressure (mPAP) in IPAH was significantly higher than CTEPH. Equally significant was the increased systolic pulmonary artery pressure (sPAP) in IPAH compared to CTEPH. Furthermore, pulmonary vascular resistance (PVR) was significantly increased in IPAH compared to patients with CTEPH. Oxygen saturation and heart rate were lower in IPAH patients than CTEPH groups. The WHO functional class major than two was significantly associated with CTEPH patients. Tricuspid annular plane systolic excursion (TAPSE) done by echocardiography, was reduced in patients with IPAH when compared to APTE. Brain natriuretic peptide (BNP) or its stable inactive pro-hormone (NT-proBNP) was higher in CTEPH than IPAH patients. Patients with APTE presented a significantly increased level of CRP and D-dimer compared to CTEPH and PAH.

### Association Between Circulating Plasma miRNA Expression, Clinical and Baseline Characteristics of the Patients

Differentially expressed miRNAs were associated with baseline and clinical characteristics of the patients, as shown in Table 3. Expression of let-7i-5p > 2.30 and miRNA-320a >2.58 was associated with APTE, high level of C-reactive protein and D-dymer. In contrast, miRNA-320a expression ≤2.58 was associated with CTEPH (*p* = 0.04) and IPAH (*p* = 0.03).

**TABLE 3:** Association between baseline and **c**linical characteristics of the patients and miRNA microarray signature.

	Dependent variables									
Clinical features	*let-7i-5p* Mean ΔCt ±SE 2.30 ± 0.74		*miR-320a* Mean ΔCt ±SE 2.58 ± 1.41		*miR-320b-1* mean ΔCt ±SE 2.07 ± 1.61		*miR-320b-2* mean ΔCt ±SE 2.34 ± 1.44		*miR-1291* mean ΔCt ±SE 2.43 ± 1.62	
	***β***	*p*	***Β***	*p*	***β***	*p*	***β***	*p*	***Β***	*p*
Age yr (median, 51)	−0.35	**0.0001**	0.48	**0.0001**	−0.41	**0.0001**	−0.44	**0.0001**	0.11	0.11
Gender		4.49								
*Female (49/60)*	−6.93	**0.0001**	−8.65	**0.0001**	−7.89	**0.0001**	−4.93	**0.0001**	4.30	**0.02**
Etiology^a^										
*APTE*	3.16	**0.00023**	4.50	**0.00028**	4.26	**0.00025**	4.29	**0.00082**	5.21	**0.00071**
*CPTEH*	1.86	0.60	−2.09	**0.04**	0.81	0.74	1.16	0.57	2.78	0.47
*IPAH*	3.85	0.39	−3.76	**0.03**	4.01	0.33	4.21	0.33	5.40	0.05
mPAH (mmHg) (mean, 45.9)	2.96	0.19	0.19	**0.0001**	3.12	0.49	3.99	0.41	4.97	0.40
sPAH (mmHg) (mean, 75.9)	−0.03	0.07	0.17	**0.0001**	2.91	0.12	3.21	0.21	4.49	0.06
PVR (dyn s cm^−5^) (mean, 349.94)	−0.001	0.16	−0.001	0.06	−0.002	0.15	0.002	0.15	−0.000026	1.00
TAPSE (mmHg) (mean, 14.50)	−0.07	0.09	−0.35	**0.0001**	−0.004	1.00	2.12	**0.0001**	3.39	**0.26**
CI (L min^−1^ m^−2^)										
1–2	−1.90	**0.009**	8.35	**0.0001**	2.13	0.67	1.98	0.83	−0.24	1.00
3–4	−0.64	**0.009**	10.17	**0.0001**	8.88	**0.0001**	6.58	**0.0001**	−0.006	1.00
WHO functional class^a^										
I vs. II	4.30	0.35	4.73	0.29	5.14	0.28	5.32	0.28	8.21	0.05
III vs. IV	3.48	0.22	3.09	0.24	3.03	1.98	2.22	0.20	4.12	0.07
CRP (mg/L) (mean, 7.63)	5.21	**0.0001**	−0.20	**0.0001**	−0.18	**0.0001**	−0.18	**0.0001**	0.07	**0.04**
D-dymer (µg/L)	4.39	**0.0001**	−0.19	**0.0001**	−0.16	**0.0001**	−0.17	**0.0001**	0.09	**0.03**

Abbreviations: APTE, acute pulmonary thromboembolism; CPTEH, chronic pulmonary thromboembolism with pulmonary hypertension; IPAH, idiopathic pulmonary hypertension; mean pulmonary arterial pression sPAH, systolic pulmonary arterial pressure; PVR, pulmonary vascular resistance; TAPSE, tricuspid annular plane systolic pressure; CI, cardiac index; CRP, C-reactive protein. General Linear Model was carried out to analyze the effect of several dependent variables on clinical features of the subjects. *β* = *β* coefficient. *P* = *P* value.

^a^FDR statistical test (statistical method for correcting the p value for multiple tests) was carried out to compare independent variables between two groups.

Bolded values are statically significant.

## Discussion

### Rationale of the Study

We described five highly expressed circulating plasma miRNAs present in APTE, CTEPH and IPAH assembled in one conglomerate. APTE, CTEPH and IPAH are rather different pulmonary vascular diseases in terms of clinical presentation, prevalence, pathophysiology, and prognosis, making it difficult to extrapolate the results among them. However, incorporating miRNA panel into baseline and clinical characteristics of the patients, we provide additional information about outcome evolution (APTE and CTEPH) and the disease form (CTEPH and IPAH), which may have implications in life-threatening cardiopulmonary disease, and specific surgical or medical treatment. To our knowledge it is an innovative study suggesting a differential spectrum of evolution and form of the disease among APTE, CTEPH and IPAH.

Among the miRNA 5-signature, let-7i-5p and miR-320a were the most expressed and functionally enriched by cancer, glioma, and PI3K-Akt signaling pathways. The network construction for target genes showed 11 genes regulated by let-7i-5p and 20 genes regulated by miR-320a. These genes regulated by let-7i-5p and miR-320a are modulators of PAAF, PAEC, and PASMC, respectively involved in proliferation, vasoconstriction, inflammation, and DNA damage. These target genes regulated by differentially expressed miRNAs were mainly enriched in GO terms and KEGG pathways. AR (androgen receptor), a target gene of hsa-let-7i-5p and has-miR-320a, was enriched in pathways in cancer, whereas PRKCA (Protein kinase C Alpha), also a target gene of hsa-let-7i-5p and has-miR-320a, was enriched in KEGG pathways, such as pathways in cancer, glioma, and PI3K-Akt signaling pathway.

Taken together, the interaction between genes and their miRNA may play important roles in the time of evolution and disease form in pulmonary hypertensive and thromboembolic diseases, as discussed below.

### Outcome Evolution in Pulmonary Thromboembolism (APTE and CTEPH)

Our results demonstrated that miR-let-7i-5p expression was upregulated in APTE and downregulated in CTEPH. Furthermore, these upregulated miRNAs in APTE were significantly enriched in cancer, glioma, and PI3K-Akt signaling pathways, whereas the downregulated miRNAs observed in CTEPH were mainly enriched in cancer and apelin signaling pathways.

Yang’s group showed that 24 miRNAs were upregulated, and 22 miRNAs were downregulated (including miR-let-7i-5p) in CTEPH compared with APTE (Miao et al., 2017). Likewise, Guo et al. also found that miR-let-7i-5p was downregulated in CTEPH patients when compared to healthy controls, as well as miR-140-3p, miR-93, miR-22, miR-106b; and that miR-1260, miR-602, miR-129-5p, miR-1908 and miR-483-5p were upregulated (Guo et al., 2014). As a tumor suppressor or tumor promoter, miR-let-7b has been found to halt cell proliferation, adhesion, and invasion by targeting Protein Code Gene (*PKA1*), Diaphanous Related Formin 2 (*DIAPH2*), Radixin (*RDX*)*, RAS, MYC,* and High Mobility Group A2 (*HMGA2*) genes and their respective proteins*. Let-7b* expression is also differently regulated in breast cancer and the data reveal its possible role in DNA repair capacity during breast carcinogenesis (Hu et al., 2013). Similarly, miR-let-7b was shown to be significantly downregulated in osteosarcoma tissues and cell lines and the functional studies revealed that the antitumor effect of miR-let-7b was probably due to targeting and suppressing Insulin Like Growth Factor 1 Receptor (*IGF1R*) expression (Zhang et al., 2019). Wang et al. demonstrated that miR-let-7b binds the 3′-UTR of Angiopoietin (*ANGPT2*) to induce migration and tube formation of Human Umbelical Vein Endothelial Cells (HUVECs), and Human Cardiac Progenitor Cells (*hCPFs*)-exosome transport to endothelial cells expressing relatively low amounts of miR-let-7b, promoting angiogenesis through upregulation of Angiopoietin 2 (*ANGPT2*) (Wang et al., 2015).

MiR-let-7i-5p has been reported as pivotal in regulating cell proliferation and migration. In fact, investigations have demonstrated that PASMCs excessively proliferate and migrate to the intima layer of pulmonary artery after APTE, remodeling the pulmonary vasculature and increasing pulmonary vascular resistance (Douma et al., 2010; Goldhaber et al., 2012; Konstantinides et al., 2014). Thus, we hypothesize that upregulation of miR-let-7i-5p may be intimately associated with excessive proliferation and migration of PASMCs following APTE which may evolve to CTEPH. Ham et al. showed that the levels of miR-let-7i-5p were upregulated in APTE compared with CTEPH, thus suppressing apoptosis via caspase in HUVECs (Ham et al., 2015).

In this way, we have also shown that 11 genes were a direct target of miR-let-7i-5p, which upregulation modulates the proliferation of human PASMCs. Among them, Interleukin13 (*IL13*) upregulation induces PASMCs in the pulmonary artery through signal transducer and activator of transcription STAT6 or through phosphatidylinositol 3-kinase (PI3K), STAT3 and mitogen activated protein kinase (MAPK) pathways (Yuan, 2019). Oxidant, arginase 2 (*ARG2*) and hypoxia-inducible factor 1α (*HIF1*) are also involved in the proliferation of PASMCs (Yuan, 2019). The experimental Schistosomiasis-associated PAH potentially depends on upregulation of *IL-13*, which can activate TGF-β1 via phospho-STAT6 signaling, or via increased matrix metalloproteinase-inducing muscular hypertrophy and periadventitial fibrosis in pulmonary arteries (Graham et al., 2010). The human eukaryotic initiation factor *EIF2C1* gene downregulates protein synthesis in response to amino acid starvation, hypoxia, and viral infection but up-regulated specific stress response proteins. *EIF2C1* expression is increased in smooth muscle cells in the vessel wall and interstitial tissue (Chen and Jiang, 2018). Toll-Like Receptor four Signaling (*TLR4*) has a prominent functional impact on platelet activity, hemostasis, and thrombosis (Vallance et al., 2017). Bone morphogenetic protein-4 (*BMP4*) acts downstream of *HIF-1* and mediates hypoxia-induced up-regulation of transient receptor potential canonical (*TRPC*), leading to increased basal [Ca2+]i in pulmonary arterial smooth muscle cells (PASMCs), promoting chronic hypoxia-induced pulmonary hypertension pathogenesis (Wang et al., 2015).

The up-regulation in miR-let-7i-5p observed only in APTE and the downregulation of miR-let-7i-5p observed only in CTEPH reinforces the abnormal remodeling between APTE and CTEPH. These differences may be associated with time of evolution, since differences were observed in cardiac index, C-reactive protein and D-dimer.

### Disease Form in Pulmonary Artery Hypertension (CTEPH and IPAH)

IPAH is an isolated small-vessel disease comprising vasoconstriction, remodeling, and thrombosis of distal vessels. On the other hand, CTEPH is characterized by large-vessel occlusion with a concomitant small-vessel disease following thromboembolism event. The pulmonary microvascular disease as a common denominator of IPAH and CTEPH probably plays a major role in pathophysiology (Berger et al., 2011; Koudstaal et al., 2020).

In the current study, we carried out NGS analysis and detection of miRNAs to understand the key miRNAs associated with disease form in CTEPH and IPAH. We found that let-7i-5p and miR-320a were down-regulated in CTEPH, while let-7i-5p was up-regulated in IPAH. Furthermore, 20 target genes were coregulated by miR-320a and other miRNAs. Androgen receptor (*AR*), a target gene of hsa-miR-320a, was enriched in cancer pathways. Protein kinase C Alpha (*PRKCA*), also a target gene of hsa-let-7i-5p and has-miR-320a, was enriched in 14 of 18 KEGG pathways, such as in cancer, glioma, and PI3K-Akt signaling pathway.

Recent studies have shown that miRNA dysregulation plays an important role in the hyperproliferative and apoptosis-resistant phenotype of pulmonary vascular cells in PH, including PAAF, PAEC, and PASMC respectively involved in proliferation, vasoconstriction, inflammation, and DNA damage (Santos-Ferreira et al., 2020). Previous studies have also uncovered that dysregulation of miRNAs such as miR-204 and miR-21 was associated with the pathobiology of IPAH (Courboulin et al., 2011; Rhodes et al., 2013; Bienertova-Vasku et al., 2015), suggesting that miRNAs may configure novel therapeutic targets. In this line, the downregulation of miR-let-7i-5p has also been observed elsewhere (Wang et al., 2013). While miR-let-7i-5p was downregulated in CTEPH, it was upregulated in IPAH and this inverse association may be crucial to differentiate both disease forms. As a tumor promoter, miR-let-7b has been found to enhance cell proliferation, adhesion, and invasion by targeting the genes of *PKA1*, *DIAPH2*, *RDX*, *RAS*, *MYC*, and *HMGA2* and respective proteins, creating an unbalance between hyperproliferative and apoptosis-resistant phenotype of PAAF, PAEC and PASMC in IPAH.

Moreover, in the present study, we demonstrated that 20 genes were a direct target of miR-320a. It has been reported these miRNA regulates cell proliferation, differentiation, and invasion in human colon cancer (Sun et al., 2012), and erythroid differentiation (Mittal et al., 2013) as well as bladder carcinoma (Shang et al., 2014) and when upregulated, miRNA-320a act toward proliferation of human PASMCs. Among the 20 target genes, a family of water-selective membrane channels promoting endothelial cell migration and angiogenesis (aquaporin—*AQP*) was found to be overexpressed in PAH patients, indicating its potential role in the treatment of PH (Saadoun et al., 2005; Schuoler et al., 2017; Gräf et al., 2018).

Considering the above evidence, we inferred that unbalance between the hyperproliferative and apoptosis-resistant phenotype of PAAF, PAEC and PASMC in pulmonary artery confer differences in IPAH and CTEPH about the diseases form. The incorporation of miRNA-320a and the unbalance of miR-let-7i-5p in baseline added by clinical characteristics signatures reinforce differences between CTEPH and IPAH.

## Conclusion

We concluded that circulating plasma miRNA and clinical/hemodynamic characteristics provide additional predictive information about the evolution outcome and the form of pulmonary thromboembolic and hypertensive diseases.

## Data Availability

We have completed the deposition of our BioProject database in a public, community-supported repository, under the number PRJNA690230 which can be accessible with the following link: http://www.ncbi.nlm.nih.gov/bioproject/690230.
