# Supplementary material for: Circulating Plasma miRNA and Clinical/Hemodynamic Characteristics Provide Additional Predictive Information About Acute Pulmonary Thromboembolism, Chronic Thromboembolic Pulmonary Hypertension and Idiopathic Pulmonary Hypertension
Source: Front Pharmacol. 2021 May 28;12:648769. doi: 10.3389/fphar.2021.648769 (PMC8194827; doi:10.3389/fphar.2021.648769)
Supplement: Supplementary file 1 [file Table1.DOCX]

**Supplementary Table S1.** Identified miRNAs in PHTD

| **miRNA ID** | **logFC** | ***P*-value** |
| --- | --- | --- |
| miR-let-7i | 3,142526117 | 0,000258666 |
| miR-320-b1 | 4,235614409 | 0,000274273 |
| miR-320a | 4,474665298 | 0,00030539 |
| miR-1291 | 5,203980549 | 0,000716661 |
| miR-320-b2 | 4,265489484 | 0,000913909 |
| miR-320-c1 | 4,501917652 | 0,002010815 |
| miR-320-c2 | 4,79877378 | 0,002719396 |
| miR-7641 | 4,019102037 | 0,010967376 |
| miR-671 | 5,258797739 | 0,012478405 |
| miR-744 | 3,586958226 | 0,013680349 |
| miR-1307 | 2,274551086 | 0,018730472 |
| miR-let-7b | 1,513143126 | 0,019192602 |
| miR-4446 | 4,445179171 | 0,022436476 |
| miR-3180-2 | 4,252129813 | 0,022829278 |
| miR-378a | 2,487294986 | 0,02305119 |
| miR-7515 | 4,197735379 | 0,027568091 |
| miR-185 | 4,604049247 | 0,028541063 |
| miR-8053 | -2,762258759 | 0,041200581 |
| miR-483 | 3,377540253 | 0,044054632 |
| miR-328 | 2,949896056 | 0,044598726 |
| miR-134 | 3,500357031 | 0,046934808 |
| miR-492 | 3,8393178 | 0,051703936 |
| miR-6723 | 3,876398895 | 0,067941954 |
| miR-378i | 3,470633248 | 0,075485961 |
| miR-500a | 3,60503169 | 0,076930367 |
| miR-106b | 2,312268894 | 0,077818537 |
| miR-3180-1 | 3,525992223 | 0,082589867 |
| miR-193b | -2,856298286 | 0,086493066 |
| miR-487a | -2,559937478 | 0,087636917 |
| miR-375 | 3,245933564 | 0,095384587 |
| miR-140 | 1,138551755 | 0,119807936 |
| miR-342 | -1,286942202 | 0,130359322 |
| miR-148b | 1,671892194 | 0,134733493 |
| miR-3200 | 2,770670965 | 0,13854888 |
| miR-887 | 2,080078323 | 0,141125154 |
| miR-222 | 1,271000075 | 0,144540544 |
| miR-149 | -1,324291384 | 0,1640338 |
| miR-6499 | -3,294701407 | 0,167370316 |
| miR-20b | 3,299973757 | 0,168889424 |
| miR-6788 | 2,95358652 | 0,173377753 |
| miR-28 | 1,803060166 | 0,18270472 |
| miR-30-b | 0,824949339 | 0,190431863 |
| miR-664-a | 1,960306207 | 0,215344797 |
| miR-361 | -0,964520196 | 0,220993742 |
| miR-7706 | 2,807466403 | 0,228721229 |
| miR-433 | 2,162633122 | 0,24235104 |
| miR-10-b | -0,68690438 | 0,242709807 |
| miR-362 | 1,995623617 | 0,243949689 |
| miR-1249 | 2,739675301 | 0,244718448 |
| miR-379 | -0,725490574 | 0,247749106 |
| miR-589 | 2,735305032 | 0,255026286 |
| miR-873 | -2,173892858 | 0,257523515 |
| miR-652 | 1,900052366 | 0,263100669 |
| miR-4278 | 2,377660279 | 0,282497932 |
| miR-95 | -2,022912387 | 0,288227592 |
| miR-99b | -0,646943963 | 0,28877201 |
| miR-339 | 2,900622102 | 0,291377726 |
| miR-100 | -0,613224465 | 0,291708423 |
| miR-6087 | 1,594644651 | 0,295760695 |
| miR-484 | 1,53487261 | 0,297963932 |
| miR-574 | 2,127507071 | 0,299497906 |
| miR-let7-fi | 0,578887244 | 0,300204645 |
| miR-146-a | 1,589234404 | 0,301163189 |
| miR-138-1 | -1,392980631 | 0,304781623 |
| miR-584 | 1,041014876 | 0,309646283 |
| miR-29-b2 | 1,153364189 | 0,320593592 |
| miR-139 | -1,01714208 | 0,320892564 |
| miR-151-a | 0,667804055 | 0,323507559 |
| miR-335 | -1,324777774 | 0,327406119 |
| mir-93 | 0,925604817 | 0,334638077 |
| miR-15b | -1,028563404 | 0,343430996 |
| miR-582 | 0,944280868 | 0,343845434 |
| miR-194-1 | -2,766762325 | 0,347671542 |
| miR-29-b1 | 0,605842984 | 0,349441231 |
| miR-135-a2 | -1,377707799 | 0,350352533 |
| miR-let7-f2 | 0,452438043 | 0,350432227 |
| miR-92-a1 | 0,4943485 | 0,366833384 |
| miR-323a | 1,6060025 | 0,380219489 |
| miR-1225 | -1,358616583 | 0,392344694 |
| miR-3615 | 1,552500703 | 0,393244777 |
| miR-210 | -1,171469058 | 0,399339828 |
| miR-126 | 0,50335838 | 0,400714784 |
| miR-129-1 | 1,031372555 | 0,406338911 |
| miR-101-1 | -0,453434881 | 0,41261566 |
| miR-1299 | 1,705003089 | 0,418709771 |
| miR-221 | 0,559380318 | 0,421199795 |
| miR-363 | -1,779143454 | 0,429290072 |
| miR-431 | 1,271516565 | 0,437198928 |
| mir-34b | -2,137806779 | 0,439390721 |
| mir-let-7d | 0,552620497 | 0,439999524 |
| miR-3074 | 1,384535639 | 0,456469154 |
| miR-345 | 2,106486167 | 0,471858233 |
| miR-1908 | 1,696995221 | 0,472370926 |
| miR-151b | 2,319557848 | 0,478101633 |
| miR-421 | 0,739670608 | 0,487342776 |
| miR-182 | -0,754592908 | 0,487555674 |
| miR-1468 | 2,137685303 | 0,491614209 |
| miR-2110 | 2,137685303 | 0,491614209 |
| miR-1224 | 1,880755751 | 0,491614209 |
| miR-590 | 1,747025339 | 0,491614209 |
| miR-365-a | 1,743525228 | 0,491614209 |
| miR-204 | -0,417781339 | 0,497676814 |
| miR-197 | 0,789506968 | 0,507324585 |
| miR-let-7a3 | 0,359611198 | 0,511869156 |
| miR-577 | 0,382506595 | 0,517538906 |
| miR-192 | 0,501930073 | 0,523396738 |
| miR-218-2 | -0,906016048 | 0,525301982 |
| miR-184 | 1,26572494 | 0,527374622 |
| miR-30d | 0,344404539 | 0,531970234 |
| miR-497 | -0,723290865 | 0,547259042 |
| miR-454 | -0,886963496 | 0,557628587 |
| miR-485 | -0,876385189 | 0,564761361 |
| miR-5189 | 1,394469575 | 0,570886411 |
| miR-16-1 | 0,342262281 | 0,580827149 |
| miR-let-7c | 0,319154938 | 0,58470787 |
| miR-941-4 | 1,610800985 | 0,587653041 |
| miR-539 | 1,09913572 | 0,595524463 |
| miR-34-c | -0,820487502 | 0,596358486 |
| miR-654 | -0,854753612 | 0,596580257 |
| miR-323b | -0,582962917 | 0,598584013 |
| miR-136 | -0,569432182 | 0,600482469 |
| miR-324 | 1,21766905 | 0,600739541 |
| miR-3168 | 0,410572523 | 0,600857327 |
| miR-142 | 0,817326766 | 0,60229115 |
| miR-592 | -0,947964073 | 0,603406158 |
| miR-212 | -1,358272469 | 0,603760548 |
| miR-381 | 0,491069423 | 0,604420433 |
| miR-181-d | 0,534633906 | 0,613567473 |
| miR-29c | -0,586092829 | 0,618153911 |
| miR-641 | -1,973856996 | 0,624947542 |
| miR-320-d2 | -1,338623477 | 0,624947542 |
| miR-628 | -0,896526331 | 0,624947542 |
| miR-941-5 | 1,357294665 | 0,626128351 |
| miR-29-a | 0,361595665 | 0,629477046 |
| miR-501 | -0,729548191 | 0,643693534 |
| miR-92-b | 0,267926838 | 0,650903955 |
| miR-769 | 0,264241151 | 0,65110264 |
| miR-877 | 0,795757321 | 0,656975721 |
| miR-98 | -0,231338631 | 0,657915985 |
| miR-124-2 | -0,333654833 | 0,658507804 |
| miR-9-2 | 0,20230212 | 0,660760297 |
| miR-143 | 0,208339877 | 0,666605519 |
| miR-205 | -2,183939482 | 0,671032414 |
| miR-191 | 0,270220241 | 0,675849938 |
| miR-26B | -0,204953322 | 0,679612485 |
| miR-9-3 | 0,201701772 | 0,680014276 |
| miR-432 | 1,093400371 | 0,695309468 |
| miR-127 | 0,522507114 | 0,701230525 |
| miR-186 | -0,422783575 | 0,70672212 |
| miR-181-a2 | 0,163473085 | 0,710893344 |
| miR-9-1 | 0,166242786 | 0,713720307 |
| miR-409 | 0,433734203 | 0,71373363 |
| miR-374-a | 0,386523516 | 0,716076046 |
| miR-19-b2 | 0,541856254 | 0,721536259 |
| miR-148-a | 0,319839808 | 0,726672291 |
| miR-340 | -0,189534377 | 0,729573514 |
| miR-195 | 0,419834139 | 0,733373749 |
| miR-128-2 | 0,260386166 | 0,740075267 |
| miR-125-a | -0,160348968 | 0,755686919 |
| miR-135-a1 | -0,858753969 | 0,761447557 |
| miR-137 | -0,330700344 | 0,762140545 |
| miR-22 | 0,235290958 | 0,780775635 |
| miR-132 | -0,187039958 | 0,784007111 |
| miR-4521 | 0,714045863 | 0,785342698 |
| miR-let-7g | 0,157848803 | 0,786015448 |
| miR-25 | 0,260343694 | 0,789010315 |
| miR-92-a2 | 0,143441975 | 0,799102431 |
| miR-128-1 | 0,22554709 | 0,800732509 |
| miR-30-e | -0,141726202 | 0,810423061 |
| miR-15a | -0,529960598 | 0,814388506 |
| miR-425 | -0,236665924 | 0,817928119 |
| miR-129-2 | 0,366109683 | 0,831877579 |
| miR-153-2 | -0,119807021 | 0,845412318 |
| miR-let-7-a1 | 0,102793681 | 0,852200831 |
| miR -101-2 | -0,12529273 | 0,857182439 |
| miR-488 | -0,838705223 | 0,85877278 |
| miR-874 | -0,128637174 | 0,859066055 |
| miR-34-a | 0,509594019 | 0,859383997 |
| miR-153-1 | 0,542997303 | 0,859875633 |
| miR-30-c2 | -0,140088501 | 0,867610334 |
| miR-326 | 0,320727295 | 0,869610188 |
| miR-19-b1 | 0,331243036 | 0,875535117 |
| miR-150 | -0,514882158 | 0,875952407 |
| miR-26-a2 | 0,071397359 | 0,877102948 |
| miR-331 | 0,199241533 | 0,87884394 |
| miR-let-7a2 | -0,072393944 | 0,880925147 |
| miR-let-7e | -0,111238157 | 0,880948907 |
| miR-181-a1 | 0,060258798 | 0,883294753 |
| miR-125-b1 | 0,08716567 | 0,883799316 |
| miR-660 | -0,584343366 | 0,883998013 |
| miR-181b2 | 0,117246428 | 0,886950571 |
| miR-301a | 0,696221746 | 0,897204004 |
| miR-17 | 0,585265692 | 0,90000623 |
| miR-3607 | -0,236988297 | 0,901511732 |
| miR-3613 | 0,805919252 | 0,907598503 |
| miR-1296 | 0,881795191 | 0,907714591 |
| miR-1301 | 0,784922361 | 0,908341952 |
| miR-219A1 | 0,874635464 | 0,908463365 |
| miR-1180 | 0,872039398 | 0,908629864 |
| miR-766 | 1,171690426 | 0,909311855 |
| miR-215 | 0,762414361 | 0,909846762 |
| miR-6716 | 0,507626919 | 0,909846762 |
| miR-7-1 | -0,090977995 | 0,911173548 |
| miR-124-3 | 0,118853575 | 0,91209501 |
| miR-99A | -0,21480595 | 0,925815692 |
| miR-411 | 0,066026294 | 0,92697686 |
| miR-504 | 2,00012514 | 0,933333333 |
| miR-382 | 1,846065649 | 0,933333333 |
| miR-370 | 1,893989415 | 0,933333333 |
| miR-31 | 1,707291043 | 0,933333333 |
| miR-3180-3 | 1,692508987 | 0,933333333 |
| miR-629 | 1,619765604 | 0,933333333 |
| miR-1237 | 1,619765604 | 0,933333333 |
| miR-18A | 1,619765604 | 0,933333333 |
| miR-6511-b2 | 1,547523068 | 0,933333333 |
| miR-548K | 1,44807229 | 0,933333333 |
| miR-1244-4 | 1,44807229 | 0,933333333 |
| miR-543 | 1,416200105 | 0,933333333 |
| miR-941-3 | 1,32820776 | 0,933333333 |
| miR-3126 | 1,583873535 | 0,933333333 |
| miR-23A | -0,155587848 | 0,940525738 |
| miR-369 | -0,389040062 | 0,944197719 |
| miR-181-b1 | 0,042779339 | 0,951132321 |
| miR-23b | -0,126890582 | 0,95189269 |
| miR-124-1 | -0,043908074 | 0,963492008 |
| miR-146b | 0,031372584 | 0,969286866 |
| miR-30-c1 | -0,145401878 | 0,972636361 |
| miR-21 | 0,043706091 | 0,977968901 |
| miR-889 | 0,115331891 | 0,979570995 |
| miR-145 | 0,2279518 | 0,980023787 |
| miR-26-a1 | 0,009866907 | 0,981606199 |
| miR-125-b2 | 0,047331284 | 0,983883791 |
| miR-30A | 0,005332461 | 0,986498442 |
| miR-181C | -0,057448428 | 0,994619499 |
| miR-6511-a4 | -1,299798284 | 1 |
| miR-365b | -1,23939842 | 1 |
| miR-6766 | -1,221125511 | 1 |
| miR-3943 | -1,208156 | 1 |
| miR-193A | 1,037746263 | 1 |
| miR-6511-b1 | 1,037524838 | 1 |
| miR-758 | 1,01709581 | 1 |
| miR-491 | 0,99586377 | 1 |
| miR-383 | -0,892197584 | 1 |
| miR-1185-2 | -0,855369842 | 1 |
| miR-152 | -0,661480326 | 1 |
| miR-1286 | -0,620443977 | 1 |
| miR-1185-1 | -0,620443977 | 1 |
| miR-1287 | -0,620443977 | 1 |
| miR-448 | 0,618954444 | 1 |
| miR-199B | 0,618954444 | 1 |
| miR-455 | 0,618954444 | 1 |
| miR-199a1 | 0,618954444 | 1 |
| miR-329-1 | 0,618954444 | 1 |
| miR-496 | 0,618954444 | 1 |
| miR-199-a2 | 0,618954444 | 1 |
| miR-770 | 0,618954444 | 1 |
| miR-767 | 0,618954444 | 1 |
| miR-7158 | 0,618954444 | 1 |
| miR-1247 | 0,618954444 | 1 |
| miR-424 | 0,603682703 | 1 |
| miR-33A | -0,602171068 | 1 |
| miR-218-1 | -0,584796396 | 1 |
| miR-299 | 0,561979247 | 1 |
| miR-133-a2 | -0,490066163 | 1 |
| miR-155 | -0,415552232 | 1 |
| miR-223 | 0,407725057 | 1 |
| miR-625 | -0,404109895 | 1 |
| miR-33b | -0,398616909 | 1 |
| miR-532 | 0,377205941 | 1 |
| miR-487b | 0,348971672 | 1 |
| miR-7-3 | -0,307034163 | 1 |
| miR-133-a1 | 0,306235826 | 1 |
| miR-708 | 0,262126559 | 1 |
| miR-4677 | -0,225261741 | 1 |
| mir-10A | -0,167237219 | 1 |
| miR-138-2 | 0,161554323 | 1 |
| miR-935 | -0,155675923 | 1 |
| miR-19A | -0,154976328 | 1 |
| miR-130a | 0,137193294 | 1 |
| miR-32 | -0,136141062 | 1 |
| miR-320-d1 | -0,123869246 | 1 |
| miR-760 | -0,10927763 | 1 |
| mir-130b | -0,106475105 | 1 |
| miR-16-2 | -0,105378133 | 1 |
| miR-24-2 | 0,095395389 | 1 |
| miR-107 | 0,067900741 | 1 |
| miR-190a | -0,066218586 | 1 |
| miR-27a | -0,053858496 | 1 |
| miR-885 | 0,046915799 | 1 |
| miR-410 | 0,044482395 | 1 |
| miR-144 | -0,044297776 | 1 |
| miR-495 | -0,024731262 | 1 |
| miR-330 | 0,018395998 | 1 |
| miR-598 | -0,004476735 | 1 |
| miR-27b | -0,002700934 | 1 |
| miR-20a | 0,002308693 | 1 |
| miR-490 | -2,56E-15 | 1 |
| miR-4460 | -2,56E-15 | 1 |
| miR-6811 | -2,56E-15 | 1 |
| POVDs: pulmonary occlusive vascular diseases; FC: fold-change; miR: microRNA. | | |
|  |  |  |
|  |  |  |
