# Supplementary material for: Circulating Plasma miRNA and Clinical/Hemodynamic Characteristics Provide Additional Predictive Information About Acute Pulmonary Thromboembolism, Chronic Thromboembolic Pulmonary Hypertension and Idiopathic Pulmonary Hypertension
Source: Front Pharmacol. 2021 May 28;12:648769. doi: 10.3389/fphar.2021.648769 (PMC8194827; doi:10.3389/fphar.2021.648769)
Supplement: Supplementary file 2 [file Table2.docx]

**Supplementary Table 2S.** Gene Ontology (GO) analysis of predicted target genes of differentially expressed miRNAs

| **GO ID** | **GO Description** | ***P*-value** | **Gene Number** | **GO Category** | **Total annotated genes** |
| --- | --- | --- | --- | --- | --- |
| GO:0005575 | Cellular_component | <1e-325 | 1379 | Cellular Component | 2,632 |
| GO:0043226 | Organelle | <1e-325 | 1074 | Cellular Component |  |
| GO:0005654 | Nucleoplasm | 2,92E-08 | 91 | Cellular Component |  |
| GO:0043234 | Protein complex | 2,48E-05 | 224 | Cellular Component |  |
| GO:0005829 | Cytosol | 9,89E-04 | 167 | Cellular Component |  |
| GO:0005815 | Microtubule organizing center | 0.02870341 | 33 | Cellular Component |  |
| GO:0001071 | Nucleic acid binding transcription factor activity | <1e-325 | 83 | Molecular Function |  |
| GO:0003674 | Molecular_function | <1e-325 | 1384 | Molecular Function |  |
| GO:0043167 | Ion binding | <1e-325 | 685 | Molecular Function |  |
| GO:0000988 | Protein binding transcription factor activity | 4,95E-04 | 61 | Molecular Function |  |
| GO:0003723 | RNA binding | 2,36E-03 | 124 | Molecular Function |  |
| GO:0019899 | Enzyme binding | 2,61E-02 | 84 | Molecular Function |  |
| GO:0000978 | RNA polymerase II core promoter proximal region sequence-specific DNA binding | 3,05E+00 | 32 | Molecular Function |  |
| GO:0042393 | Histone binding | 7,31E+00 | 23 | Molecular Function |  |
| GO:0030234 | Enzyme regulator activity | 7,38E+00 | 57 | Molecular Function |  |
| GO:0001077 | RNA polymerase II core promoter proximal region sequence-specific DNA binding transcription factor activity involved in positive regulation of transcription | 0.001008153 | 27 | Molecular Function |  |
| GO:0035198 | MiRNA binding | 0.03062485 | 7 | Molecular Function |  |
| GO:0010467 | Gene expression | <1e-325 | 52 | Biological Process |  |
| GO:0006464 | Cellular protein modification process | <1e-325 | 306 | Biological Process |  |
| GO:0009058 | Biosynthetic process | <1e-325 | 471 | Biological Process |  |
| GO:0034641 | Cellular nitrogen compound metabolic process | <1e-325 | 561 | Biological Process |  |
| GO:0048011 | Neurotrophin TRK receptor signaling pathway | <1e-325 | 53 | Biological Process |  |
| GO:0008150 | Axon guidance | 1,11E-09 | 1339 | Biological Process |  |
| GO:0038095 | Fc-epsilon receptor signaling pathway | 9,38E-06 | 29 | Biological Process |  |
| GO:0022607 | Cellular component assembly | 2,05E+00 | 125 | Biological Process |  |
| GO:0006950 | Response to stress | 3,18E-01 | 184 | Biological Process |  |
| GO:0008219 | Cell death | 9,39E-01 | 62 | Biological Process |  |
| GO:0044403 | Symbiosis, encompassing mutualism through parasitism | 6,16E+01 | 36 | Biological Process |  |
| GO:0007173 | Epidermal growth factor receptor signaling pathway | 7,41E+01 | 19 | Biological Process |  |
| GO:0016032 | Viral process | 0.0001378472 | 32 | Biological Process |  |
| GO:0008543 | Fibroblast growth factor receptor signaling pathway | 0.0001666239 | 19 | Biological Process |  |
| GO:0048015 | Phosphatidylinositol-mediated signaling | 0.000177578 | 23 | Biological Process |  |
| GO:0065003 | Macromolecular complex assembly | 0.0003527867 | 85 | Biological Process |  |
| GO:0006351 | Transcription, DNA-templated | 0.0007393064 | 150 | Biological Process |  |
| GO:0009056 | Catabolic process | 0.001049952 | 145 | Biological Process |  |
| GO:0061024 | Membrane organization | 0.001292097 | 39 | Biological Process |  |
| GO:0006397 | mRNA processing | 0.002452151 | 43 | Biological Process |  |
| GO:0061418 | Regulation of transcription from RNA polymerase II promoter in response to hypoxia | 0.002828394 | 8 | Biological Process |  |
| GO:0006367 | Transcription initiation from RNA polymerase II promoter | 0.00393423 | 20 | Biological Process |  |
| GO:1900740 | Positive regulation of protein insertion into mitochondrial membrane involved in apoptotic signaling pathway | 0.004436857 | 6 | Biological Process |  |
| GO:0006921 | Cellular component disassembly involved in execution phase of apoptosis | 0.00656308 | 7 | Biological Process |  |
| GO:0007596 | Blood coagulation | 0.008213337 | 28 | Biological Process |  |
| GO:0006461 | Protein complex assembly | 0.009058384 | 46 | Biological Process |  |
| GO:0044281 | Small molecule metabolic process | 0.01018041 | 164 | Biological Process |  |
| GO:0034655 | Nucleobase-containing compound catabolic process | 0.01211897 | 52 | Biological Process |  |
| GO:0043687 | Post-translational protein modification | 0.01293754 | 11 | Biological Process |  |
| GO:0000278 | Mitotic cell cycle | 0.01693828 | 23 | Biological Process |  |
| GO:0006488 | Dolichol-linked oligosaccharide biosynthetic process | 0.02727785 | 6 | Biological Process |  |
| GO:0007268 | Synaptic transmission | 0.03068918 | 17 | Biological Process |  |
| GO:0030203 | Glycosaminoglycan metabolic process | 0.04107096 | 10 | Biological Process |  |
| GO:0006112 | Energy reserve metabolic process | 0.04108693 | 10 | Biological Process |  |
