# Supplementary material for: Circulating Plasma miRNA and Clinical/Hemodynamic Characteristics Provide Additional Predictive Information About Acute Pulmonary Thromboembolism, Chronic Thromboembolic Pulmonary Hypertension and Idiopathic Pulmonary Hypertension
Source: Front Pharmacol. 2021 May 28;12:648769. doi: 10.3389/fphar.2021.648769 (PMC8194827; doi:10.3389/fphar.2021.648769)
Supplement: Supplementary file 3 [file Table3.docx]

**Supplementary Table 3S.** The KEGG pathways

| **Pathway term** | ***P*-value** | **Gene number** |
| --- | --- | --- |
| Apelin signaling pathway | 1,11E-10 | 12 |
| Signaling pathways regulating pluripotency of stem cells | 5,21E+01 | 31 |
| Estrogen signaling pathway | 0.000136688 | 11 |
| Glioma | 0.0002054161 | 11 |
| Prostate cancer | 0.0003325287 | 16 |
| Circadian rhythm | 0.0005223237 | 10 |
| Glycosaminoglycan biosynthesis | 0.0005967453 | 4 |
| Proteoglycans in cancer | 0.001323158 | 18 |
| Hippo signaling pathway | 0.001593522 | 17 |
| AMPK signaling pathway | 0.001734528 | 18 |
| Endometrial cancer | 0.004441939 | 9 |
| Viral carcinogenesis | 0.005773483 | 22 |
| Melanoma | 0.01656659 | 12 |
| mTOR signaling pathway | 0.01908568 | 11 |
| Thyroid hormone synthesis | 0.03342257 | 2 |
| PI3K-Akt signaling pathway | 0.03654019 | 33 |
| Amoebiasis | 0.04116968 | 10 |
| Non-small cell lung cancer | 0.04216641 | 9 |
